# Thymocyte selection-associated high mobility group box gene (TOX) is aberrantly over-expressed in mycosis fungoides and correlates with poor prognosis

**DOI:** 10.18632/oncotarget.2031

**Published:** 2014-05-28

**Authors:** Yuanshen Huang, Ivan V. Litvinov, Yang Wang, Ming-Wan Su, Ping Tu, Xiaoyan Jiang, Thomas S. Kupper, Jan P. Dutz, Denis Sasseville, Youwen Zhou

**Affiliations:** ^1^ Molecular Medicine Lab and Chieng Genomics Centre, Vancouver Coastal Health Research Institute, Vancouver, BC, Canada; ^2^ Department of Dermatology and Skin Science, University of British Columbia, Vancouver, BC, Canada; ^3^ Terry Fox Laboratory, British Columbia Cancer Agency and Department of Medical Genetics, University of British Columbia, Vancouver, BC, Canada; ^4^ Division of Dermatology, McGill University Health Center, Montreal, QC, Canada; ^5^ Department of Dermatology and Venereology, Peking University First Hospital, Beijing, China; ^6^ Department of Dermatology, Brigham and Women's Hospital, Harvard Skin Disease Research Center, Harvard University, Boston, MA, USA; ^7^ Dermatologic Oncology Program, British Columbia Cancer Agency, Vancouver, BC, Canada

**Keywords:** TOX, mycosis fungoides, cutaneous T cell lymphoma, marker, prognostic factor

## Abstract

Mycosis fungoides (MF) often mimics the common chronic inflammatory skin diseases and is difficult to be diagnosed with certainty, partly because of the lack of well-characterized molecular markers. Previously, we discovered that *TOX*, a key T cell development regulator,was aberrantly over-expressed in early stage MF. In the current multi-center study involving two independent patient cohorts, we determined the prevalence of *TOX* over-expression in the full spectrum of MF skin biopsies, and tested if *TOX* expression levels correlated with long term clinical outcomes. We examined *TOX* expression levels in 113 MF biopsies. We found that the MF biopsies expressed higher *TOX* mRNA than the controls in both cohorts (17.9 fold in cohort 1, *P* = 0.002; 5.8 fold in cohort 2, *P* < 0.0001). In addition, thicker skin lesions such as plaques and tumors expressed even higher *TOX* levels than thinner patches. Further, *TOX* over-expression differentiated MF from the controls (area under the curve [AUC]=0.87, *P* < 0.0001). Finally, high *TOX* mRNA levels correlated with increased risks of disease progression (*P* = 0.003) and disease-specific mortality (*P* = 0.008). In conclusion, *TOX* may be a useful marker for improving MF diagnosis and prognostication.

## INTRODUCTION

The incidence of cutaneous T cell lymphoma (CTCL) is 6.4 per million and rising, making it the second most common extranodal non-Hodgkin lymphomas [1, 4]. Characterized by clonal accumulation of post-thymic T cells residing in the skin [2], CTCL represents a group of diseases that are heterogeneous in clinical presentation and prognosis. The most common variants are mycosis fungoides (MF) and Sézary syndrome (SS), two distinct but related diseases, together representing more than 80% of CTCL[2-4]. In the early disease stages, which can last several years, MF presents as flat erythematous skin patches resembling benign inflammatory dermatoses (BID), such as allergic contact dermatitis, eczema, or psoriasis; whereas in the later stages, MF cells gradually form plaques or tumors, and may disseminate to the lymph nodes and internal organs. The cancerous cells in some patients may appear in the peripheral blood, a hallmark of the leukemic stage of CTCL[2].

At present, it is difficult to accurately diagnose MF. The current approach combines clinic-pathological features in three categories: compatible clinical presentations, compatible pathologic features, and presence of T cell receptor (TCR) gene clonal rearrangements (International Society of Cutaneous Lymphomas ISCL Criteria, 2005) [5]. However, these features are not universally or exclusively present in MF. This is particularly true for early stage MF (eMF), which has clinical, histological, and even molecular (including TCR clonality) resemblance to common benign inflammatory skin diseases [5-8]. Further, eMF skin lesions contain only a small number of malignant cells admixed with a large number of reactive CD4^+^ T cells. As a result, diagnostic delays in MF are common, which can occasionally exceed a decade.

Another challenge in MF management is to predict long term clinical outcome. While most patients with eMF have a life span similar to that of healthy individuals, 9% of MF patients with limited patch and/or plaque disease, as well as 24% of patients with extensive patches or plaques, will experience disease progression. MF patients with end-stage disease have a high mortality [9, 10]. It is therefore critical to identify MF patients with high risk of disease progression or disease-specific mortality. However, few robust prognostic markers exist for MF, which hinders the accurate prediction of disease prognosis.

In a recent study focusing on gene markers of eMF, we identified 19 genes that were highly enriched in eMF skin biopsies, compared with benign control biopsies. The most enriched gene in eMF biopsies is *TOX*[11], a critical nuclear factor regulating thymocyte lineage commitment. The aberrant *TOX* expression in MF was subsequently confirmed in early as well as more advanced disease of MF by in a small patient population (N=15)[12]. These studies raised the possibility that TOX could be used as a marker for MF diagnosis and prognostication.

The aim of the current study is to test this possibility. We examined the expression level of *TOX* in 113 MF skin biopsies from two independent MF cohorts, including 59 patients for whom long term clinical outcome data, such as disease progression and survival, are available. We found that *TOX* was aberrantly over-expressed in the majority of MF skin lesions in both cohorts, and that higher TOX expression levels correlated with increased risks of disease progression and disease-specific mortality.

## RESULTS

### Demographics of study subjects

A total of 149 individuals contributed skin biopsies, including 113 with MF (summarized in Table 1), 25 with BID, and 11 with healthy skin (HS).

**Table 1:** Demographics and clinical characteristics of patients with MF (N=113)

Demographics or Characteristics	Cohort 1 (N=54)	Cohort 2 (N=59)
Sex		
Male	40 (74%)	36 (61%)
Female	14 (26%)	23 (39%)
Age at diagnosis (years)		
Median (range)	50 (28-85)	62 (26-91)
Race		
Caucasian	17 (31%)	59 (100%)
Chinese	31 (57%)	
Other	6 (11%)	
Clinical stage		
I	31 (57%)	42 (71%)
II	16 (30%)	4 (7%)
III	5 (9%)	5 (8%)
IV	2 (4%)	8 (14%)

### *TOX* is ectopically over-expressed in MF skin biopsies

**Figure 1:**
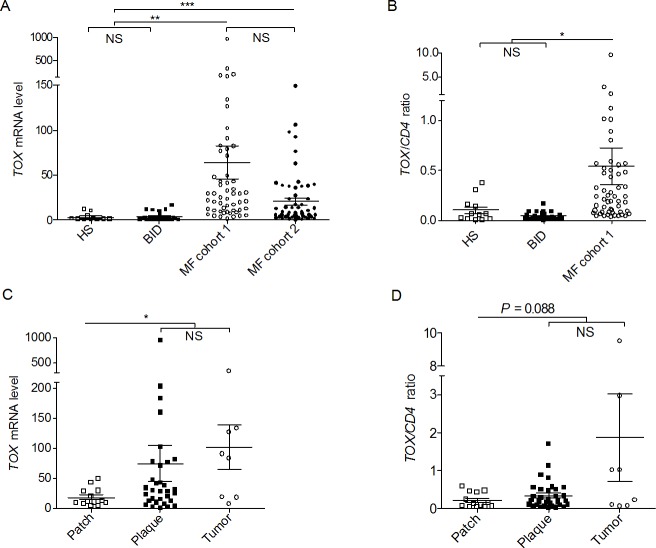
*TOX* mRNA levels are increased in MF skin biopsies (A) *TOX* mRNA in MF samples compared with BID and HS. **, *P* = 0.002, ***, *P* < 0.0001. (B) The ratios of *TOX* mRNA/*CD4* mRNA in samples of MF, BID and HS. *, *P* = 0.011. (C) *TOX* mRNA in different types of MF lesions. *, *P* = 0.018. (D) The ratios of *TOX* mRNA/*CD4* mRNA in different types of MF lesions. Horizontal bars denote the mean and standard error of mean for each sample type analyzed. Two tailed t tests were used for comparison. NS=not significant.

First, *TOX* mRNA expression in skin biopsies was assessed in cohort 1patients, using HS and BID biopsies as the controls. Since the levels of *TOX* mRNA expression (mean ± standard error of the mean) of BID were not significantly different from those of HS (3.77 ± 0.94 versus 3.15 ± 1.24, *P* = 0.70), they were combined to form the non-MF controls (3.58 ± 0.75). Compared with this, MF cohort 1 had a 17.9 fold increase in *TOX* gene expression (64.21 ± 18.69, *P* = 0.002) (Figure 1A).

To evaluate if the *TOX* over-expression in MF skin biopsies is the result of simple increase of CD4^+^ T cells in MF skin biopsies compared with benign controls, *TOX* mRNA level was normalized to CD4 mRNA level in the same skin biopsies. In Figure 1B, *TOX* mRNA to CD4 mRNA ratios were also much higher (8.7 fold) in MF samples, compared with non-MF controls (0.542 ± 0.182 versus 0.062 ± 0.014, *P* = 0.011).

Next, to test if *TOX* expression varies with tumor burden, *TOX* mRNA levels were plotted according to lesional morphological types, such as patches, plaques, and tumors. As shown in Figure 1C, skin samples from thicker skin lesions of MF, including plaques and tumors, expressed higher levels of *TOX* (74.91 ± 29.72 in plaques and 101.3 ± 37.23 in tumors)*,* compared with thinner patches (18.31 ± 4.10, *P* = 0.018). Similarly, *TOX*/*CD4* ratios displayed an increase in thicker lesions (0.348 ± 0.065 in plaques and 1.870 ± 1.145 in tumors), compared with thinner lesions (0.216 ± 0.055 in patches, *P* = 0.088, Figure 1D).

To further confirm the *TOX* mRNA expression increase in MF skin lesions, an independent MF cohort (cohort 2) was analyzed. As shown in Figure 1a, *TOX* mRNA in cohort 2 was also much higher than that in non-MF controls (20.64 ± 3.90 versus 3.58 ± 0.75, *P* < 0.0001).

### *TOX* protein is detected in the CD4^+^ T cell nuclei in various MF skin lesions and its levels are in parallel with lesional thickness

**Figure 2:**
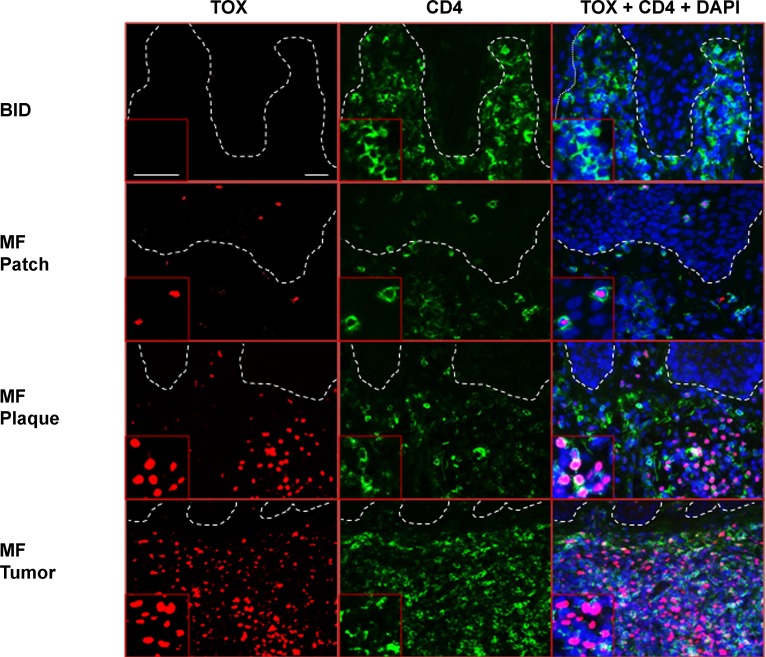
Ectopic *TOX* protein is detected in CD4^+^ T lymphocytes in MF skin lesions, but absent in BID BID (shown here is CD) and MF skin biopsies were stained with antibodies against TOX (red, Alexa Fluor® 594) and CD4 (green, Alexa Fluor® 488). DAPI was used to stain the nuclei of cells. Insets: magnification from representative areas. Bars=20 μm.

To test if TOX protein can be detected in the CD4^+^ T cells of MF lesions, we performed immunofluorescence (IF) staining on patch, plaque, and tumor MF biopsies, using benign chronic dermatitis (CD) lesions as the controls. In line with the *TOX* mRNA expression results, although the CD skin biopsies contained numerous CD4^+^ T cells, few of them had detectable TOX protein expression. In contrast, strong nuclear staining of TOX was detected in the CD4^+^ T cells of MF lesions. In addition, thicker MF skin lesions ­(plaque and tumor types) displayed stronger TOX staining than patch lesions (Figure 2).

### High *TOX* mRNA levels differentiate MF from non-MF skin biopsies

**Figure 3:**
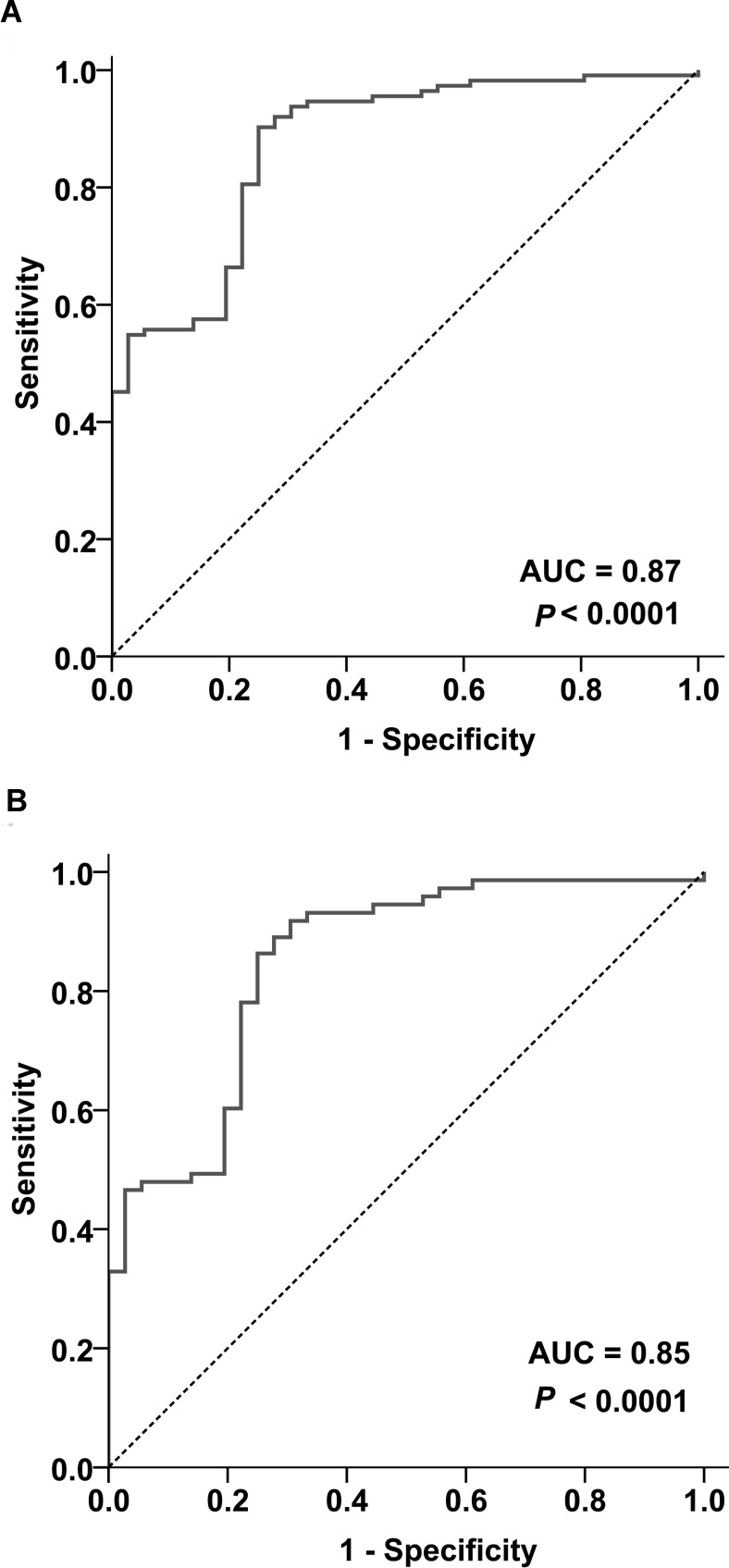
Increased *TOX* mRNA levels differentiate MF from non-MF biopsies (A) ROC analysis on the MF biopsies as a whole (N=113) and non-MF skin biopsies (N=36). (B) ROC analysis on the stage-I MF biopsies (N=73) and non-MF skin biopsies (N=36). AUC=Area under the curve.

We next applied receiver operating characteristic (ROC) curve to evaluate if increased *TOX* mRNA levels could differentiate MF from non-MF controls. As shown in Figure 3, *TOX* mRNA levels had good discriminatory power for MF, demonstrated by an area under the curve (AUC) value of 0.87 (95% confidence interval (CI) =0.81-0.94, *P* < 0.0001, Figure 3A). When cutoff was set at 2.99, *TOX* mRNA levels had 90.3% sensitivity and 75.0% specificity for MF.

Given that early MF is most difficult to diagnose in the clinical practice, ROC curve was further plotted in stage-I MF (N=73). *TOX* mRNA levels had an AUC of 0.85 (95% CI= 0.77-0.93, *P* < 0.0001, Figure 3B) for stage-I MF, with a sensitivity of 86.3% and a specificity of 75.0% at the cutoff of 2.99.

### High *TOX* mRNA levels define a group of MF patients with increased risks of disease progression and disease-specific mortality

**Figure 4:**
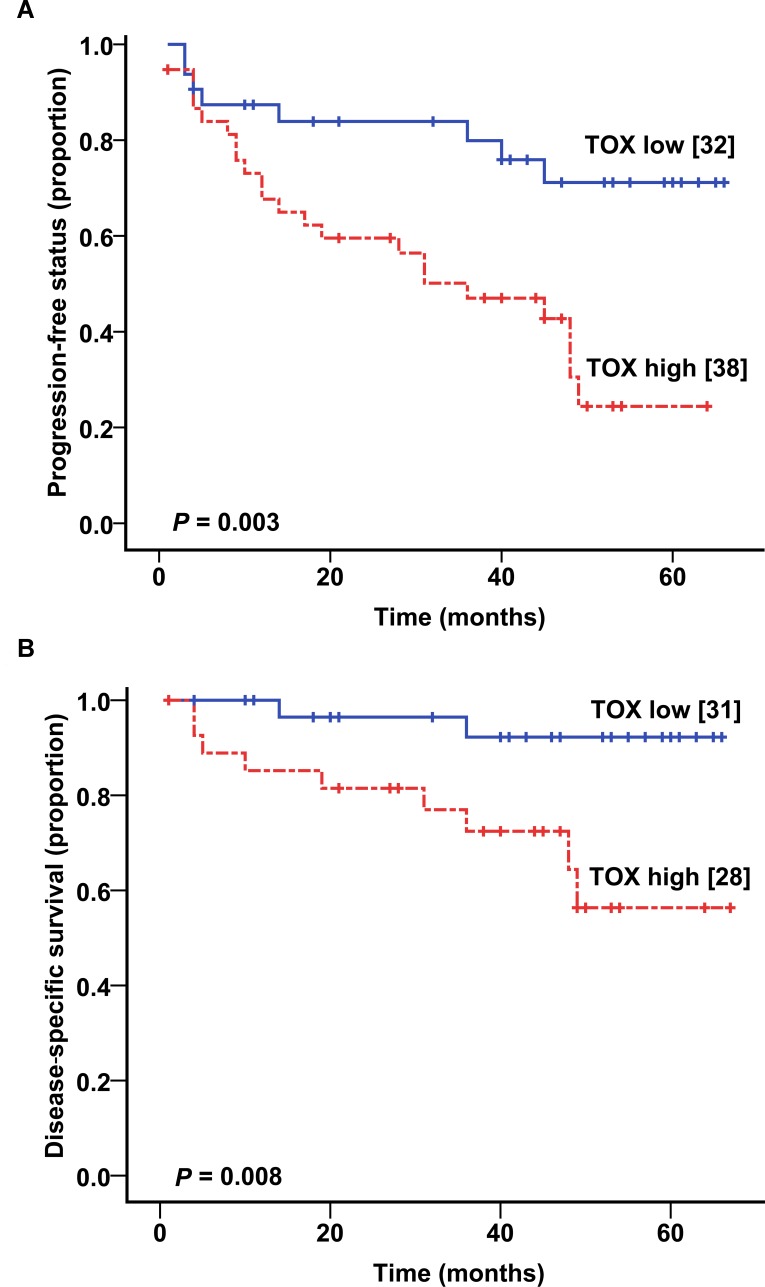
Higher *TOX* mRNA levels correlate with worse clinical outcome (A) Kaplan-Meier plot showing the relationship between *TOX* mRNA levels and disease progression in MF patients. (B) The relationship between *TOX* mRNA levels and disease-specific mortality in MF patients.

To test if *TOX* expression in MF skin biopsies influences disease progression and disease-specific mortality, we evaluated MF cohort 2, for whom clinical outcome data are available for up to 6 years (median follow up time = 45 months). Kaplan-Meier curves showed that high *TOX* mRNA levels in skin biopsies were associated with much increased likelihood of disease progressing into a higher clinical stage during the follow up period (*P* = 0.003, Figure 4A). Meanwhile, those cases with low *TOX* expression had very low tendency to progress clinically during the follow up period. Through multivariate testing, we found that high *TOX* expression remained significantly associated with disease progression, after adjusting for stage, age, and sex (hazard ratio (HR) = 2.62, *P* = 0.028, Table 2).

**Table 2:** Multivariate analyses of prognostic factors in MF disease progression and disease-specific mortality

Prognostic factor	Progression				Disease-specific mortality		
	HR	95.0% CI	P		HR	95.0% CI	P
TOX	2.62	1.1 to 6.2	0.028		6.16	1.2 to 30.8	0.027
Stage	3.57	1.6 to 7.9	0.002		5.31	1.2 to 23.5	0.028
Age	1.02	1.0 to 1.0	0.246		1.00	1.0 to 1.0	0.989
Sex	1.02	0.5 to 2.3	0.960		0.55	0.1 to 2.1	0.386

In addition, high *TOX* mRNA levels were strongly associated with increased disease-specific mortality (*P* = 0.008, Figure 4B) in MF patients. Multivariate analysis showed that high *TOX* mRNA expression was an independent predictor of worse survival after adjusting for other variables (HR = 6.16, *P* = 0.027, Table 2), including clinical stage, age and sex.

Collectively, these findings supported that high *TOX* mRNA levels defined a subset of MF patients with increased risks of disease progression and disease-specific mortality.

## DISCUSSION

The earliest evidence of aberrant *TOX* expression in MF came from our previous transcriptome analysis focusing on eMF, which showed that eMF CD4^+^ T cells, but not CD4^+^ T cells in BID, over-expressed *TOX* at both mRNA level and protein level[11].

*TOX* encodes a nuclear protein of the high-mobility group (HMG) box superfamily. It contains a DNA-binding domain, which allows it to regulate transcription by modifying local chromatin structure and modulating the formation of multi-protein complexes [13, 14]. In mouse models, *TOX* was reported to be essential for the proper development of CD4^+^ thymocyte [13, 15]. Tightly controlled in a stage-specific manner in developing thymocytes, *TOX* is down-regulated before T cells exit the thymus, and remains suppressed in the peripheral lymphoid tissues [13]. Despite its rapid induction by pre-TCR and TCR signalling in immature thymocytes, *TOX* is not induced by TCR signalling in mature T cells[13]. Hence its up-regulation in eMF lesional CD4+ T cells is unlikely to be a result of T cell activation. Rather, this unusual observation led us to propose that *TOX* is a disease marker with diagnostic and/or prognostic value for MF.

This study tested and confirmed our hypothesis. Our findings not only confirmed *TOX* expression in eMF, but also demonstrated that *TOX* ectopic expression is a common feature across the entire spectrum of MF. Moreover, high *TOX* mRNA levels effectively differentiate MF, both as a whole and for stage-I MF alone, from benign inflammatory or healthy skin. Finally, high *TOX* expression strongly correlated with worse prognosis, including increased disease progression and disease-specific mortality.

MF clinically resembles the far more common, but clinically benign skin inflammatory diseases such as CD, psoriasis, and cutaneous reactions to drugs. Differentiating MF from these benign conditions is difficult, largely due to the lack of well-defined molecular markers in the clinical setting. Although CD2, CD3, CD5, and CD7 deficiency is included in ISCL criteria to define early MF, the loss of CD2, CD3, and/or CD5 in T cells is only 10% sensitive, despite its 100% specificity. CD7 deficiency is about 40% sensitive and 80% specific in general [5]. Therefore better markers with higher sensitivity and specificity are needed. Although a small number of markers have been reported for MF skin biopsies, including loss of *CD13*, ectopic expression of *BLK* gene, microRNAs (including miR-155, miR-203 and miR-205) [16-18], *BCL7A* loss [19, 20], enhanced *AHI1* [21], and *CD158K/KIR3DL2* in transformed advanced MF [22], few of these markers were tested in multicenter studies, or used in a clinical setting.

Similarly, achieving accurate prognostication of MF patients is challenging clinically. Several molecular markers have been described to be of potential prognostic value. For MF prognosis, persistence of the same CD4^+^ T cell clone over time in skin biopsies correlated with an aggressive disease course[23]. Loss of *BCL7A* expression predicted aggressive disease course in patients with early stage CTCL [20]. Specifically for tumor stage MF, presence of chromosomal alterations on 9p21, 8q24, 10q26qter and 1q21-1q22 often indicates a poor prognosis [24, 25]. The clinical utility of these markers needs further evaluation in larger studies.

In light of these, the current multicenter study consisting of patients with diverse ethnic origins demonstrated considerable potential of *TOX* to improve management for MF. Kaplan-Meier curve analysis demonstrated that MF lesions, including early MF lesions, that contain no or low *TOX* mRNA expression had little tendency of disease progression or MF-related death. *TOX* as a marker can identify these low-risk patients for whom conservative management perhaps would be adequate rather than being subjected to more toxic treatments, such as topical nitrogen mustard, or carmustine. In contrast, MF patients with high levels of *TOX* expression may benefit from early and more aggressive treatment to prevent disease progression and to reduce MF-related mortality.

Despite the predictive potential of *TOX* for MF demonstrated in this study, caution needs to be taken while interpreting its clinical usefulness. Due to the rarity of this disease, only a moderate sample size of 113 can be reached from three study centres from three countries. Additional confirmation in other centres is warranted to uncover the true clinical relevance of *TOX* for MF diagnosis and prognostication.

Notwithstanding this limitation, the multicenter nature of our study underscores the consistency of *TOX* up-regulation in patients from diverse ethnical and geographic backgrounds. Furthermore, *TOX* as a marker is highly robust and user-friendly, since it can be detected by a number of routine dermatopathologic tools, such as qPCR, IF, and immunohistochemistry staining.

Given *TOX*'s critical regulatory role in CD4^+^ T cell development and its aberrant over-expression in the majority of MF skin lesion, it is highly likely that *TOX* activation plays a pathogenic role in MF. Further studies are therefore warranted to evaluate if *TOX* ectopic expression contributes to the development of MF. If proven to be true, *TOX* may emerge as a novel therapeutic target for MF in the future.

In summary, *TOX* ectopic expression is readily and frequently detected in the malignant CD4^+^ T cells in MF skin biopsies, including the most challenging eMF. Moreover, increased *TOX* expression levels define a group of MF patients with increased risk of disease progression and disease-specific mortality. Therefore, characterization of *TOX* expression status of MF patients may be valuable not only for diagnostic confirmation but also for guiding MF management in the future.

## MATERIALS AND METHODS

### Study subject recruitment

With approval from institutional clinical ethics board, this study was conducted according to Declaration of Helsinki Principles, and all participants has provided their written informed consent. Two MF cohorts were examined in this study (Table 1). Cohort 1 was prospectively recruited from Vancouver Canada and Beijing China from 2008-2012, including 54 subjects with MF (cohort 1, N=54) recruited from the Skin Lymphoma Clinics of British Columbia Cancer Agency (BCCA, Vancouver, Canada, N=26), and Peking University First Hospital (Beijing, China, N=28). In addition, 25 individuals with BID (9 psoriasis, 13 CD, and 3 pityriasis rubra pilar) and 11 volunteers with HS were recruited from the outpatient dermatology clinic of University of British Columbia (UBC, Vancouver, Canada). Cohort 2 of MF patients (cohort 2, N=59) was collected from Harvard University with institutional approval and had been well-characterized previously[20, 21, 26, 27]. The diagnosis and clinical staging were established according to the diagnostic criteria of CTCL[7].

### Preparation of clinical samples and analyses of *TOX* mRNA and protein

Obtaining skin biopsies, RNA extraction, and quantitative reverse transcription-polymerase chain reaction (qPCR) were performed using protocols that were previously described [11]. Beta actin (*ACTB*) mRNA was used as the internal control. The results were expressed as copies of *TOX* mRNA per 1000 copies of *ACTB* mRNA. In addition, TOX protein in MF and control skin biopsies was analyzed by IF using specific antibodies against TOX (rabbit anti-TOX polyclonal antibody, Sigma-Aldrich, St. Louis, MO) and human CD4 protein (mouse monoclonal anti-human CD4 antibody, Dako, Denmark), as previously described [11]. Cell nuclei were counterstained with 4' 6-diamidino-2-phenylindole (DAPI). The slides were visualized under a Zeiss AxioVert 200M inverted fluorescence microscope (Carl Zeiss AG, Jena, Germany). Representative sections of the micrographs were obtained and processed with the Zeiss AxioVision 4.8 image acquisition and processing software (Carl Zeiss AG). Brightness and contrast were adjusted consistently across all images.

### Statistical analyses

SPSS 14 (Chicago, IL), GraphPad Prism 5.00 (San Diego, CA), and X-Tile (New Haven, CT) were used for statistical analyses. P values < 0.05 were considered to be statistically significant.

Continuous variables were compared by two-tailed t tests. ROC method was used to analyze the sensitivity and specificity of *TOX* mRNA levels to differentiate MF from BID or HS skin biopsies.

X-Tile software, a marker cutpoint analysis tool developed by Camp RL et al[28], was applied to determine the optimal cut-point for *TOX* expression level as 8.2 (Miller-Siegmund corrected P value=0.027). In our analysis of disease progression and disease-specific mortality, *TOX* high and *TOX* low groups were defined by the expression levels higher or lower than 8.2, respectively.

MF patients were considered to experience disease progression when disease progressed to more advanced clinical stages (i.e. beyond stage I) and/or death due to MF during the follow up period[20]. Individuals with multiple progressions (n > 1) were counted as n progression events. Survival time was defined as the duration from the date of sample collection to death.

Disease progression and disease-specific mortality rates were assessed using the Kaplan-Meier curves. Univariate analysis using the log-rank test was first used to detect any association between *TOX* mRNA level and disease progression and disease-specific mortality. Multivariate analyses using COX proportional hazards regression were then used to evaluate the following prognostic factors: stage at diagnosis (stages I and II versus stages III and IV), *TOX* mRNA level, age, and sex.

### CONFLICT OF INTEREST

The authors disclose no potential conflicts of interest.
